# At the Heart of the Diagnosis: A Case of Systemic Lupus Erythematosus Presenting as Cardiac Tamponade

**DOI:** 10.7759/cureus.34447

**Published:** 2023-01-31

**Authors:** Jose Gomez Casanovas, Mery Bartl, Laura Rincon-Rueda, Christine E Loftis, Emilia Dulgheru

**Affiliations:** 1 Internal Medicine, University of Texas Rio Grande Valley School of Medicine, Edinburg, USA; 2 Rheumatology Institute, Doctors Hospital at Renaissance, McAllen, USA

**Keywords:** sle-associated pah, pericardial window, hispanic population, systemic lupus erythema, pericardial effusion. cardiac tamponade

## Abstract

Systemic lupus erythematosus (SLE) is a heterogenous, systemic disease characterized by the production of pathogenic autoantibodies against nuclear antigens. Although the most common cardiac manifestation of SLE is pericardial effusions, their progression to cardiac tamponade is rare and has an incidence between 1-3%. We describe a case of a 42-year-old Hispanic woman who presented with severe shortness of breath, vague chest pain, and hemodynamic compromise secondary to cardiac tamponade. The patient’s underlying etiology of cardiac tamponade was attributed to a new diagnosis of SLE based on the 2019 European Alliance of Associations for Rheumatology/American College of Rheumatology classification (EULAR/ACR) criteria for SLE. The patient’s treatment consisted of a pericardial window and immunosuppressive therapy with corticosteroids, Mycophenolate, and hydroxychloroquine. This case aims to increase awareness of SLE as a possible differential diagnosis of cardiac tamponade in the appropriate clinical setting.

## Introduction

Systemic lupus erythematosus (SLE) is a heterogeneous, systemic disease characterized by the production of pathogenic autoantibodies against nuclear antigens generating multiorgan inflammation [1]. Although SLE can affect the pericardium, myocardium, valvular structures, and the conduction system, pericardial involvement is the most common [2]. Pericardial effusions are the most frequent manifestation and are typically treated conservatively with immunosuppressive therapy; however, large pericardial effusions resulting in tamponade and hemodynamic compromise can rarely occur [3]. We describe a case of a 42-year-old Hispanic woman who presented with severe shortness of breath, vague chest pain, and hemodynamic compromise secondary to cardiac tamponade. The underlying etiology of the patient's presentation was attributed to SLE. This article will discuss the pathophysiology of cardiac tamponade, describe the rarity of this presentation as a manifestation of SLE, and aims to increase awareness of SLE as a possible differential diagnosis of cardiac tamponade in the appropriate clinical setting.

## Case presentation

A 42-year-old Hispanic woman presented to the hospital with progressively worsening shortness of breath over two months. The patient's shortness of breath was exacerbated with exertion and associated with vague chest tightness and infrequent episodes of lightheadedness. Upon further questioning, the patient noticed raised, non-pruritic lesions on the anterior aspect of her chest, face, nasal bridge, hairline, and posterior neck, aggravated with exposure to the sunlight that started about two months prior to presentation. Over the previous month, the patient developed swelling to her lower extremities, pain, and swelling to the small joints of her hands with associated morning stiffness, fatigue, and dry cough. She denied symptoms of alopecia, weight loss, fever, oral or nasal ulcers, Raynaud's phenomenon, or hemoptysis. The patient’s only known past medical history was diabetes, hypertension, and dyslipidemia.

**Figure 1:**
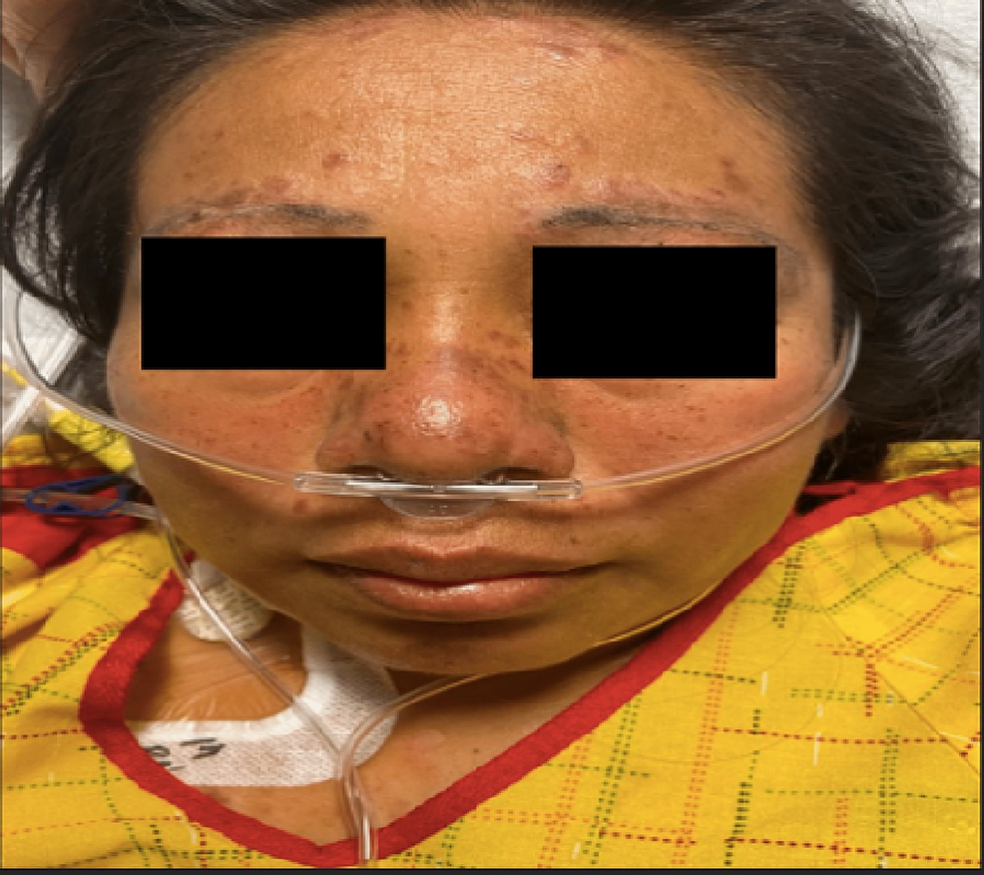
Erythematous rash noted at the anterior nasal bridge and cheeks in a butterfly distribution sparing the nasolabial folds, and several raised, hyperpigmented, round lesions noted to the hairline and eyebrows

**Figure 2:**
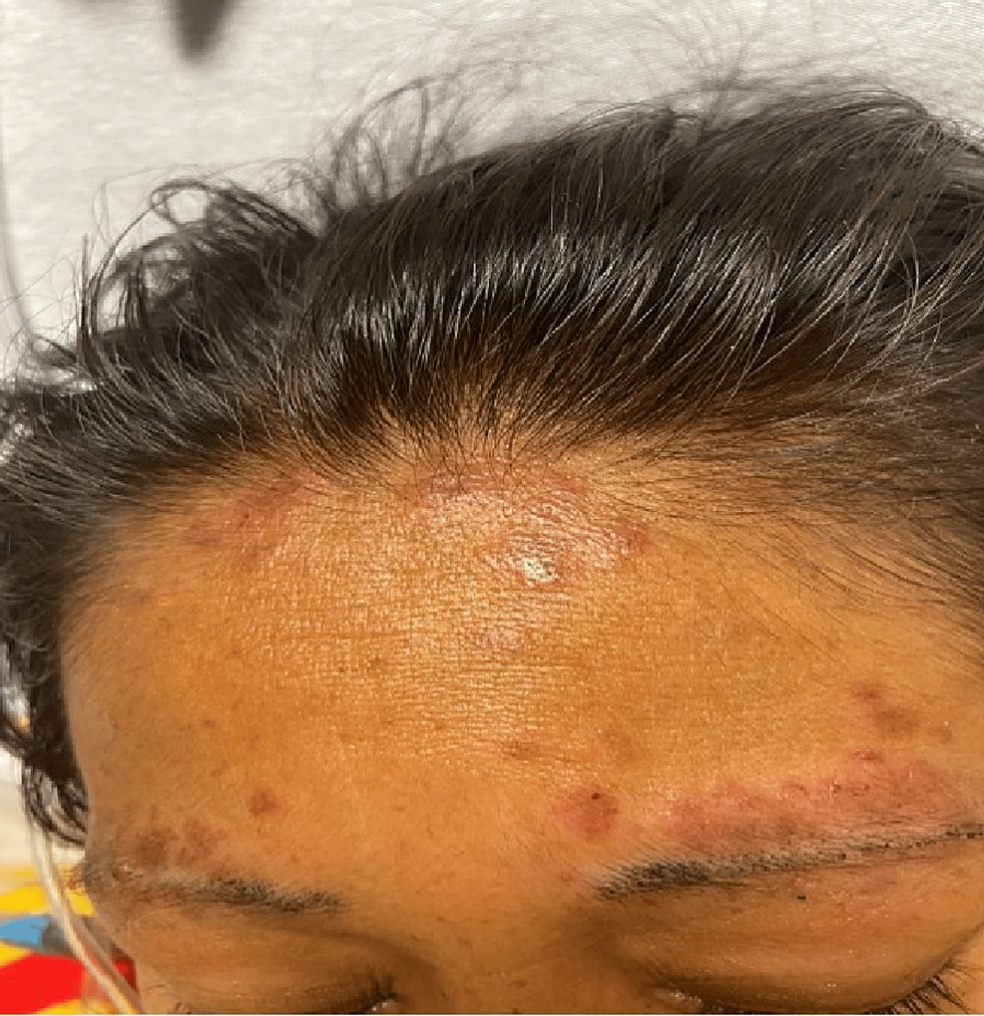
Several raised, hyperpigmented, round lesions noted on the hairline and eyebrows

On examination, the patient's vital signs showed a blood pressure of 98/68 mmHg, a heart rate of 104 beats per minute, a temperature of 99.3 degrees F, and oxygen saturation of 92% on room air. The patient was ill-appearing and was in moderate distress. She was found to have mild peri-orbital edema, an erythematous rash to the anterior nasal bridge and cheeks in a butterfly distribution sparing the nasolabial folds, and several raised, hyperpigmented round lesions noted to the hairline, eyebrows anterior aspect of the chest, posterior neck, and scalp (Figures 1-2).

The patient had an elevated jugular venous pressure of 5 cm above the sternal angle, was tachycardic with the presence of a loud midsternal pericardial friction rub, had diminished breath sounds in the lung bases bilaterally, and had 2+ pitting edema to bilateral lower extremities.

**Figure 3:**
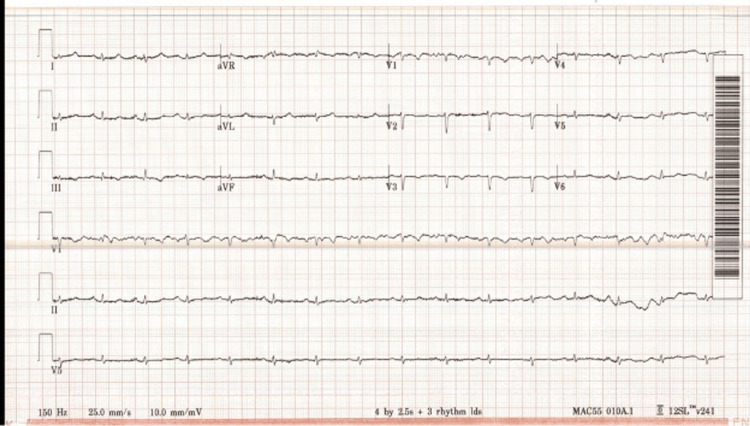
EKG demonstrating sinus tachycardia with low voltage QRS and few premature ventricular complexes

An electrocardiogram revealed sinus tachycardia with low voltage QRS and few premature ventricular complexes (Figure 3). Arterial blood gas was performed while the patient was breathing ambient air and showed a pH of 7.34, pCo2 28, pO2 62, and a bicarbonate level of 18.9.

Her laboratories revealed a white blood cell count of 2.6 cells/uL, hemoglobin 10 gm/dL with a mean corpuscular volume of 77%, and a platelet count of 186 cells/uL. The sodium level was 130 mmol/L, potassium 5.2 mmol/L, bicarbonate 16 mmol/L, blood urea nitrogen (BUN) 44 mg/dL, creatinine 1.1 mg/dL, blood glucose level 126 mg/dL, thyroid-stimulating hormone (TSH) 1.5 IU/mL, erythrocyte sedimentation rate (ESR) 62 mm/hr, C-reactive protein (CRP) 0.4 mg/dL, and an albumin level of 2.9 g/dL. Her brain natriuretic peptide (BNP) level was 224 pg/mL, and her urinalysis showed hematuria, pyuria, and proteinuria. A 24-hour urine protein to creatinine ratio demonstrated 2.7 mg/gm.

**Figure 4:**
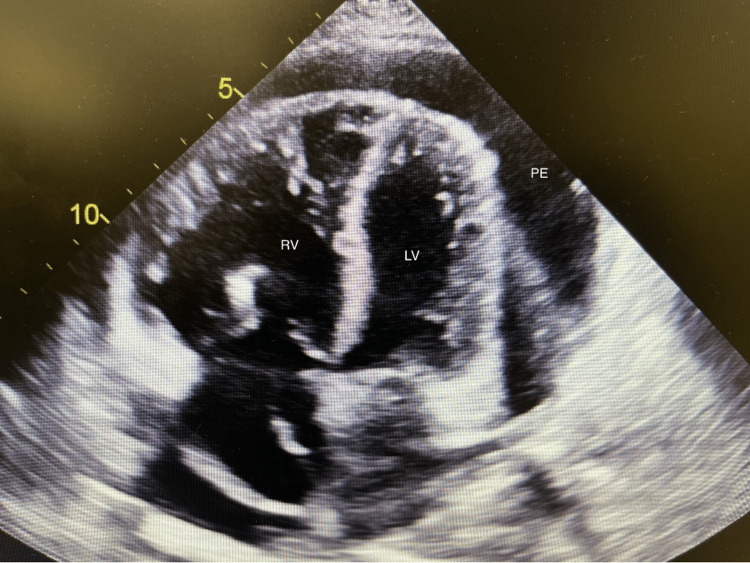
Apical view of the heart by 2D-echocardiogram with the presence of moderate to large pericardial effusion (PE) and underfilled left (LV) and right ventricle (RV) The left atrium is not shown because it collapsed.

**Figure 5:**
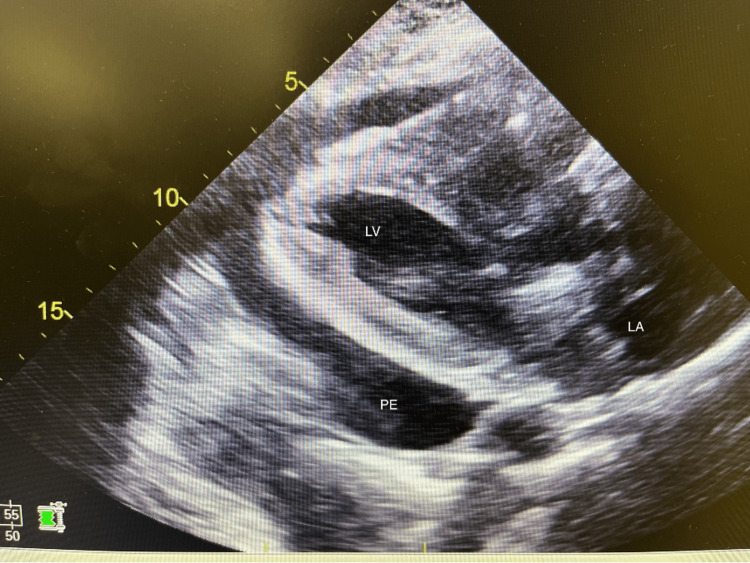
Long-axis view of the heart by 2D-echocardiogram with the confirmed presence of moderate to large pericardial effusion (PE), with clear compression of the left ventricle (LV) and left atrium (LA) collapse

Given the patient's clinical presentation and concern for cardiac tamponade, a bedside point-of-care ultrasound was performed, which revealed a large pericardial effusion, bilateral pleural effusions, and a hyperdynamic heart with an enlarged right ventricle. Subsequently, a formal cardiac echocardiogram was performed, which showed a normal left ventricular (LV) cavity with an ejection fraction of around 55% to 60%, mild LV hypertrophy, moderate to severe tricuspid regurgitation, right ventricular systolic pressure of 85-90 mmHg, and a large pericardial effusion with a respiratory variation of mitral inflow and compression of the right atrium suggesting tamponade physiology (Figures 4-5).

Because of the patient's physical examination and findings on cardiac echocardiogram, the patient underwent left anterolateral thoracotomy with pericardial window and biopsy and drainage of 700 mL of pericardial fluid that was sent for analysis. Pericardial fluid cultures revealed no bacterial, acid-fast bacilli (AFB,) or fungal growth. The fluid cytology revealed few reactive mesothelial cells but was negative for malignant cells. The pericardial biopsy revealed fibro-collagenous tissue with attenuated lining epithelium, capillaries, and inflammation without any atypia or malignancy.

Because of her initial laboratory studies and clinical presentation, an autoimmune workup was performed and revealed an antinuclear antibody (ANA) by ELISA ratio of >32, ANA by immunofluorescence of 1:1280, double-stranded DNA 706 IU/mL, anti-SS-A antibody >240 U/mL, anti-SS-B antibody 19 U/mL, complement C3 of 23 mg/dL, and complement C4 <5 mg/dL. The rest of her autoimmune workup was negative and is shown in Table 1. The diagnosis of systemic lupus erythematosus was made based on a score of 35 according to the 2019 European Alliance of Associations for Rheumatology/American College of Rheumatology (EULAR/ACR) classification criteria for SLE. Of note, the calculated Systemic Lupus Erythematosus Disease Activity Index (SLEDAI) was 32 with a score of higher than 20, representing high disease activity.

**Table 1:** Laboratory studies MCV: mean corpuscular volume; BUN: blood urea nitrogen; BNP: brain natriuretic peptide; RNP: ribonucleoprotein; ANA: antinuclear antibody; ANCA: antineutrophil cytoplasmic antibodies

Parameter	Patient	Reference Range
WBC	2.6 th/L	4.8-10.9 th/L
Hemoglobin	10.0 gm/dL	10.8-14.7 gm/dL
MCV	77 fL	84-96 fL
Platelet	186 th/uL	146-388 th/uL
Sodium	130 mmol/L	132 -143 mmol/L
Potassium	5.2 mmol/L	3.5-5.1 mmol/L
Bicarbonate	16 mmol/L	21-31 mmol/L
BUN	44 mg/dL	7-25 mg/dL
Creatinine	1.1 mg/dL	Baseline 0.9 mg/dL
BNP	225 pg/mL	>100 pg/mL
DNA ds Ab	706 IU/mL	<10 IU/mL
RNP 70	0.5 U/mL	<7.0 U/mL
ANA	>32.0 ratio	>1.0 ratio
Cardiolipin Ab IgG	<14 GPL	< or =1 4
Cardiolipin Ab IgM	<12 GPL	< or =14
B2-Glycoprotein IgM Ab	<9 GPL	< or =20
B2-Glycoprotein IgG Ab	<9 GPL	< or =20
Anti-centromere antibody	<0.4 U/mL	<7.0 U/mL
Jo 1 Antibody	<0/3 U/mL	<7.0 U/mL
Scl 70 antibody	<0.6 U/mL	<7.0 U/mL
Smith antibody (Sm)	<0.8 U/mL	<7.0 U/mL
SS-A/Ro antibody	>240 U/mL	<7.0 U/mL
SS-B/La Antibody	19 U/mL	<7.0 U/mL
U1RNP antibody	0.6 U/mL	<7.0 U/mL
Complement C3	23 mg/dL	87-200 mg/dL
Complement C4	<5 mg/dL	19-52 mg/dL
Immunoglobulin A	242 mg/dL	66-443 mg/dL
Anti-streptolysin O	<1 IU/mL	<250 IU/mL
ANCA	Negative	
Urine culture	Negative	
Random urine protein/creatinine ratio	2697 mg/gm	161 mg/gm

In addition to the pericardial window, the patient was treated with pulse methylprednisolone 500 mg daily for three days, followed by a transition to oral prednisone 40 mg three times daily (TID), mycophenolate 500 mg twice daily (BID), and hydroxychloroquine 200 mg daily. Despite drainage of the pericardial effusion and improvement of pleural effusions with immunosuppressive therapy, the patient continued to have shortness of breath. Due to findings of elevated right ventricular systolic pressure, the diagnosis of pulmonary hypertension was entertained. The patient underwent right heart catheterization, which revealed wedge pressure of 25 mmHg, mean pulmonary arterial pressure of 25 mmHg, and pulmonary vascular resistance of 3.22 Woods units, confirming the diagnosis of pulmonary hypertension with a World Health Organization (WHO) Class I and II overlap.

The patient was started on sildenafil 40 mg daily with titration up to 80 mg TID and furosemide 40 mg daily with a moderate improvement of symptoms. Given the complexity of her initial presentation, the patient's kidney biopsy was deferred and was scheduled to be performed in the outpatient setting, but she continued mycophenolate 1.0 gm BID along with hydroxychloroquine 200 mg daily. The patient was discharged with close follow-up with rheumatology, cardiology, nephrology, and pulmonology teams.

## Discussion

Cardiac tamponade is a known medical emergency characterized by fluid accumulation in the pericardium, which results in restriction of the filling of the cardiac chambers, and if not treated promptly, can lead to cardiac arrest [4]. Infectious, inflammatory, and neoplastic processes can all result in pericardial effusions. The rate and acuity at which these fluids accumulate within the pericardial space are the primary drivers for the development of tamponade physiology. 

The pericardium is a membranous tissue surrounding the heart, and its primary function is to protect and reduce the mechanical friction. The pericardial cavity contains approximately 15-50 ml of ultra-filtrated plasma between its visceral and parietal layers. Pericardial inflammation or pericarditis can present as acute, chronic, or as pericardial effusion. Although the pericardium has some degree of elasticity, sudden or constantly increasing accumulation of fluid in the pericardial space may compromise the heart’s ability to distend and can lead to external compression of the heart. As a result of this compression, the hemodynamics are compromised, resulting in decreased compliance of the ventricles and subsequently decreased venous return and cardiac output.

Although pericardial effusions frequently occur in patients with SLE and typically present clinically as pericarditis, cardiac tamponade is rare and occurs in less than 1-3% of patients [3]. The low incidence of cardiac tamponade in SLE may be related to the fact that patients typically present with other overt manifestations such as arthritis, rashes, or cytopenias, and incidentally, smaller effusions are detected earlier in the disease course [5]. One observational study found that more than 50% of patients with a pericardial effusion had concomitant mucocutaneous manifestations [6]. The presence of a rash or oral ulcers would likely prompt patients to seek medical care sooner, which would expedite testing and diagnosis resulting in earlier treatment and fewer complications such as cardiac tamponade.

According to the literature, patients with SLE who present with cardiac tamponade typically complain of shortness of breath, chest pain, hypotension, and will have raised jugular pressure [6]. Moreover, there have been several studies performed to try to determine which patients are at risk for progression to cardiac tamponade. One retrospective study found that patients with pericardial effusions who developed tamponade had a statistically lower complement C4 level compared to those that did not develop tamponade physiology [7]. Furthermore, a more recent retrospective study found that the presence of pleuritis and the anti-nucleosome antibody positivity are significant predictors of progression to cardiac tamponade in patients with SLE. Regardless of risk factors, patients who develop tamponade physiology have a poor prognosis with one study, noting a 46% survival at the five-year follow-up [8].

Since cardiac tamponade is a medical emergency, the goal of treatment aims to promptly evacuate pericardial fluid in order to improve cardiac function. After removal of fluid by either pericardiocentesis or pericardiotomy, prevention of re-accumulation is best achieved by the combination of intravenous glucocorticosteroids plus or minus the addition of an immunosuppressive agent such as cyclophosphamide [6,9].

## Conclusions

Cardiac tamponade is a rare complication of SLE and occurs in 1-3% of patients with SLE. A high index suspicion of underlying autoimmune disease as the etiology of cardiac tamponade should be warranted in young reproductive-aged women with systemic symptoms, including serositis, rashes, arthralgias, and fatigue. Our patient’s gender, age, clinical presentation, and laboratory findings suggested that her underlying etiology was likely secondary to an autoimmune disease. Typically, patients with pericardial effusions can be treated with conservative immunosuppressive management; however, it is essential to note that because cardiac tamponade is a medical emergency, and regardless of etiology, prompt treatment should be with a pericardial window or pericardiocentesis to decrease disease-related complications.
